# Shoulder Damage Model and Its Application for Single Point Diamond Machining of ZnSe Crystal

**DOI:** 10.3390/ma15010233

**Published:** 2021-12-29

**Authors:** Shenxin Yin, Huapan Xiao, Wenjun Kang, Heng Wu, Rongguang Liang

**Affiliations:** 1 College of Aerospace Engineering, Chongqing University, Chongqing 400044, China; yinshenxin@foxmail.com; 2 College of Mechanical and Vehicle Engineering, Chongqing University, Chongqing 400044, China; 3 College of Optical Sciences, University of Arizona, Tucson, AZ 85721, USA; wkang1@email.arizona.edu (W.K.); heng.wu@foxmail.com (H.W.); rliang@optics.arizona.edu (R.L.)

**Keywords:** diamond machining, ZnSe crystal, subsurface damage, undeformed chip thickness

## Abstract

The damaging of ZnSe crystal has a significant impact on its service performance and life. Based on the specific cutting energies for brittle and ductile mode machining, a model is proposed to evaluate the damage depth in the shoulder region of ZnSe crystal during single point diamond machining. The model considers the brittle-ductile transition and spring back of ZnSe crystal. To verify the model, the elastic modulus, hardness, spring back, and friction coefficient of ZnSe crystal are measured by nanoindentation and nanoscratch tests, and its critical undeformed chip thickness is obtained by spiral scratching. Meanwhile, orthogonal cutting experiments are conducted to obtain the different shoulder regions and cutting surfaces. The shoulder damage depth is analyzed, indicating that the effect of the feed on the damage depth at a high cutting depth is stronger than that at a low one. The model is verified to be effective with an average relative error of less than 7%. Then, the model is used to calculate the critical processing parameters and achieve a smooth ZnSe surface with a roughness Sa = 1.0 nm. The model is also extended to efficiently predict the bound of the subsurface damage depth of a cutting surface. The research would be useful for the evaluation of surface and subsurface damages during the ultra-precision machining of ZnSe crystal.

## 1. Introduction

Single point diamond turning (SPDT) has high efficiency, precision, and repeatability and is broadly used to fabricate high-precision parts in the fields of optics, clean energy, information communication, etc. [1]. Particularly in near-infrared and infrared applications, SPDT is gradually playing a non-negligible role in prototyping crucial optical elements [2]. As a striking infrared crystal, ZnSe is widely used for CO_2_ lasers and night vision systems. However, owing to its high brittleness, different forms of damage, such as cracks and pits, arise inevitably in SPDT, greatly limiting the service performance and life of the fabricated ZnSe optical elements [3]. To achieve a damage-free ZnSe surface, it is essential to firstly investigate the generation process and quantitative characterization of the damages to a diamond-machined ZnSe surface.

Many researchers have studied the generation process of micro-cracks or pits for SPDT-processed brittle crystal materials, which are closely related to the damages in the shoulder region. Zhang et al. [4] revealed that plastic flows and brittle cracks coexisted in the shoulder region. The median crack produced subsurface damages (SSDs) and the lateral crack determined the surface damages. Zong et al. [5] believed that SSD would be generated only when the median crack in the brittle-ductile transition (BDT) zone approached the cutting surface of ZnS crystal. However, Wang et al. [6] hold that SSD would appear when any median cracks in the shoulder region penetrated beneath the cutting surface. Interestingly, they found that this would not happen in the actual fly cutting of KDP crystal. Zhang et al. [7] proposed a new viewpoint for the diamond-turned KDP crystal: the effects of all median cracks at the right side of the BDT zone on a cutting surface could be ignored when the tool rake angle was negative, but it would be no longer effective when the tool rake angle was zero. Some discrepancies exist among the previous research, which may be attributed to different material properties or processing parameters. It is necessary to study the generation process of damages specifically for diamond-machined ZnSe. Naturally, the damages in the shoulder region should be effectively calculated.

Damage depth is the main parameter to quantitatively characterize the defects in brittle crystal materials, especially the SSD depth. Most researchers have used destructive methods to measure the SSD depth [8,9,10,11]. For example, Yan et al. [9] utilized a focused ion beam to prepare a thin single-crystal silicon sample and then measured the depth of subsurface line defects with a cross-sectional transmission electron microscopy. Tie et al. [11] used the deliquescent magnetorheological finishing spot method to measure the depth of a subsurface defect in KDP crystal. These methods are direct and reliable, and therefore can precisely provide the damage information, but they are time-consuming and may modify the physical properties of the tested sample. Consequently, the sample may not be reused, which raises the production cost. Therefore, some non-destructive methods are proposed. For example, Yan et al. [12,13] proposed that the amorphous deformed layer depth of silicon could be calculated by the ratio of the Raman intensity of total amorphous silicon to that of total crystalline silicon. With the same method, Lai et al. [14] evaluated the subsurface amorphous deformed layer in crystalline germanium. These methods are fast and convenient, but the equipment and instruments involved are generally expensive. In the above destructive and non-destructive methods, the prepared sample is generally very small compared to the whole optical component, so only localized areas can be inspected, and the measured results are limited. Therefore, some theoretical models are put forward to calculate the SSD depth. Blackley et al. [15] developed a BDT model to evaluate the depth of the median crack in germanium. To acquire the equivalent depth of the median crack in diamond-turned Cleartran ZnS crystal, Zong et al. [16] employed the Vickers indentation model, but the model was limited to calculating the median crack depth at the BDT zone. Yu et al. [17] developed a machining model to determine the SSD depth by analyzing the surface damage region for the microstructured surface in a brittle material, but the measurement of the damage region was random. Our previous research has also proposed a theoretical model to calculate the SSD depth of ZnSe crystal in diamond cutting [18]. Nevertheless, the maximum calculation error is too high, up to about 20.0%. In the above-mentioned models, the damage information for the whole component is not involved. An optical element often requires multi-step cutting processes. The damages caused by the current step must be removed or relieved by the next step. This makes it valuable to predict the bound of the SSD depth for a component.

In this paper, based on the specific cutting energies for brittle and ductile mode machining, a model of the shoulder damage is developed for diamond-processed brittle material. The material properties and critical undeformed chip thickness (UCT) of ZnSe are measured, and several groups of the cutting surface and shoulder region are machined. The shoulder damage model is validated, and its application is discussed. This study will provide an important reference for the nanodefect-free machining of ZnSe optical elements.

## 2. Shoulder Damage Model

In this paper, the shoulder damage model is developed and used to study the generation process of damages and to predict the bound of the SSD depth in diamond-machined ZnSe. SPDT is a complex machining process with a large number of parameters. It is almost impossible to develop a model by considering all of the factors. Consequently, it is necessary to make some simplifications and assumptions in the modeling, which are as follows:

(i) The tool wear effect is ignored, and its vibration amplitude is stable at all speeds;
(ii) The movement error of the workpiece spindle is ignored;
(iii) The deformations of the tool and the workpiece are not considered.

**Figure 1:**
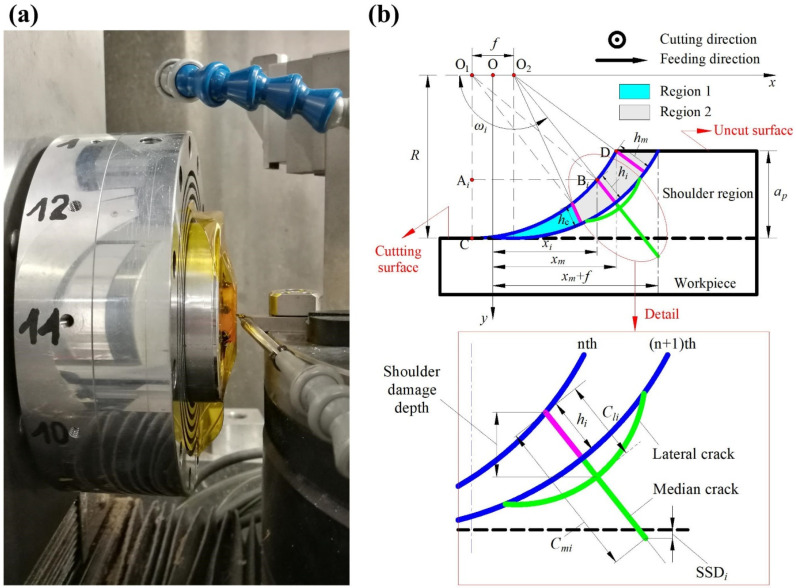
(**a**) Experimental setup and (**b**) cutting geometry characteristics (bottom right: detailed view of the crack configuration in shoulder region) of SPDT. Region 1: plastic flow region; region 2: brittle crack region.

Figure 1a,b show the experimental setup and cutting geometry characteristics of SPDT, respectively [18]. Points O_1_ and O_2_ are the trajectory centers of the nth and (*n* + 1)th cuts, respectively. The origin O of the coordinate system *x*O*y* is located in the middle of points O_1_ and O_2_. The instantaneous UCT *h_i_* increases from zero at point C (the tool nose tip) to a maximum value *h_m_* at point D along the circular arc of the tool nose. Additionally, *x_i_* and *x_m_* are the *x* coordinates at *h_i_* and *h*_*m*_, respectively. According to the indentation fracture mechanics, the median and lateral cracks may appear in SPDT. There is a critical UCT *h_c_* separating the cutting area into regions 1 (plastic flow region) and 2 (brittle crack region). The material in region 1 is cut in ductile mode, and the material in region 2 is cut in brittle mode. The UCT *h_c_* occurs at the BDT zone. The UCT *h_i_* can be estimated by [7]:

(1) $$h_{i}=\left\{ \begin{array}{l} \frac{f}{R}x_{i}\;\;\;\;\;\;\;\;\;\;\;\;\;\;\;\;\;\;\;\;\;\;\;\;0\leq x_{i}\leq x_{m}\\ \frac{x_{m}(x_{m}+f-x_{i})}{R}\;\;\;\;\;\;\;\;\;\;\;\;x_{m}<x_{i}\leq x_{m}+f \end{array}\right.$$

(2) $$x_{m}=\sqrt{R^{2}-{\left(R-a_{p}\right)}^{2}}-\frac{f}{2}$$

where *R* is the nose radius of cutting tool; *f* and *a_p_* are the feed and cutting depth, respectively.

The UCT *h_i_* increases from zero at the beginning. At this point, it is smaller than the cutting edge radius of the cutting tool. The equivalent rake angle *α_e_* is related to the UCT *h_i_* and cutting edge radius *r*, which can be expressed as [19]:

(3) $$\alpha_{e}=\left\{ \begin{array}{l} \sin^{-1}\left[\frac{h_{i}}{r}-1\right]\;\;\;\;\;\;\;\;\;\;\;\;h_{i}\leq r(1+\sin\alpha )\\ \alpha \;\;\;\;\;\;\;\;\;\;\;\;\;\;\;\;\;\;\;\;\;\;\;\;\;\;\;\;\;\;\;\;\;\;\;h_{i}>r(1+\sin\alpha ) \end{array}\right.$$

where *α* is the nominal rake angle.

In the micro/nanoscale cutting process, the equivalent shear angle *φ_e_*, depending on the UCT *h_i_*, can be calculated by [7,20]:

(4) $$\varphi_{e}=\left\{ \begin{array}{l} \tan^{-1}\left(\frac{r_{c}\cos\alpha_{e}}{1-r_{c}\sin\alpha_{e}}\right)\;\;\;\;\;\;\;\;\;\;\;\;\;h_{i}\leq r(1+\sin\alpha )\\ \varphi =\frac{\pi }{4}-\frac{\beta }{2}+\frac{\alpha }{2}\;\;\;\;\;\;\;\;\;\;\;\;\;\;\;\;\;h_{i}>r(1+\sin\alpha ) \end{array}\right.$$

where *r_c_* is the cutting ratio; *φ* is the nominal shear angle; and *β* is the friction angle. Here, *β* = arctan *μ_f_*, and *μ_f_* is the friction coefficient at the tool flank face. Venkatachalam et al. [19] have given three levels of *r_c_* as 0.25, 0.3, and 0.35, and three levels of *μ_f_* as 0.2, 0.3, and 0.4 for micro machining of a single-crystal brittle material. Zhang et al. [7] and Arif et al. [20] used *r_c_* = 0.3 for the diamond cutting of KDP crystal, single crystal silicon, and BK7 glass. In this paper, *r_c_* is empirically configured as 0.3, and *μ_f_* is obtained by experiments. 

Arif et al. [21] have analyzed the BDT phenomenon by the specific cutting energies for brittle and ductile mode machining. The cutting energy in ductile mode is related to the tangential cutting force and cutting speed. Arcona et al. [22] have derived the equation for the tangential cutting force *F_c_*:

(5) $$F_{c}=\frac{HA}{3}\left(\frac{\cot\varphi_{e}}{\sqrt{3}}+1\right)+\mu_{f}\sigma_{f}A_{f}$$

where *H* is the workpiece hardness; *A* is the undeformed chip area; *σ_f_* is the stress at the flank-workpiece interface; and *A_f_* is the contact area of the tool-workpiece at the flank-workpiece interface.

The undeformed chip area *A* is expressed as [22]:

(6) $$A=R^{2}\theta -(R-h_{i})\sqrt{h_{i}(2R-h_{i})}$$

where *θ* is the included sector angle of the tool nose at *h_i_*, which can be derived by [4]:

(7) $$\theta =\tan^{-1}\frac{\sqrt{h_{i}(2R-h_{i})}}{R-h_{i}}$$

The stress *σ_f_* is related to the material properties of the workpiece, which is given by [22]:

(8) $$\sigma_{f}=k_{1}H\sqrt{\frac{H}{E}}$$

where *k*_1_ is a parameter; *E* is the elastic modulus of the workpiece.

The contact area *A_f_* is determined by [21]:

(9) $$A_{f}=W\left(\frac{2s}{3\tan\theta_{f}}\right)$$

where *W* is the width of the contact area at the flank-workpiece interface; *s* is the spring back; and *θ_f_* is the clearance angle.

The width *W* is calculated by [22]:

(10) $$W=2\sqrt{h_{i}(2R-h_{i})}$$

The spring back *s* is calculated using Equation (11) [23]:

(11) $$s=k_{2}h_{i}$$

where *k*_2_ is the spring back coefficient, which is obtained by the experiments in this paper.

Taking Equations (6)–(11) into Equation (5), the equations for the cutting energy *E_d_* and specific cutting energy *E_sp_*_−*d*_ in ductile mode machining are respectively [4]:

(12) $$E_{d}=F_{c}\times v=\left[\frac{HA}{3}\left(\frac{\cot\varphi_{e}}{\sqrt{3}}+1\right)+2\mu_{f}\sqrt{h_{i}(2R-h_{i})}\left(\frac{2s}{3\tan\theta_{f}}\right)\left(k_{1}H\sqrt{\frac{H}{E}}\right)\right]v$$

(13) $$E_{sp-d}=\frac{F_{c}}{A}=\frac{\frac{HA}{3}\left(\frac{\cot\varphi_{e}}{\sqrt{3}}+1\right)+2\mu_{f}\sqrt{h_{i}(2R-h_{i})}\left(\frac{2s}{3\tan\theta_{f}}\right)\left(k_{1}H\sqrt{\frac{H}{E}}\right)}{R^{2}\theta -(R-h_{i})\sqrt{h_{i}(2R-h_{i})}}$$

The material in brittle mode machining is removed by the interference of cracks in the shoulder region. The fracture energy *E_f_* expended in brittle mode is associated with the crack surface area and specific surface energy, which can be calculated by [24]:

(14) $$E_{f}=(2\pi C_{l}+2C_{m})v\gamma_{S}$$

where *γ_S_* is the specific surface energy (the energy per unit area required to break the bonds), *γ_S_* = 1.0 J/m^2^ [25]; *C_m_* and *C_l_* are the mean depths of the median and lateral cracks in the shoulder region, respectively, which can be expressed as:

(15) $$C_{l}=average(C_{li}),C_{m}=average(C_{mi})$$

where *C_li_* and *C_mi_* are the depths of the lateral and median cracks at *h_i_*, respectively; the function *average* is to obtain the mean value of crack depths at all *h_i_* from *h_c_* to *h_m_*. The relationship between *C_li_* and *C_mi_* can be expressed as [24]:

(16) $$C_{mi}=k_{3}C_{li}$$

where *k*_3_ is a dimensionless parameter depending on the indenter system.

The material removal volume *V_b_* in the median/lateral crack system is calculated by [4]:

(17) $$V_{b}=0.5\pi C_{l}^{2}v$$

In brittle mode machining, the plastically deformed enclave still exists, and the equations for the cutting energy *E_b_* and specific cutting energy *E_sp_*_−*b*_ are, respectively [21],

(18) $$E_{b}=E_{f}+E_{d}$$

(19) $$E_{sp-b}=\frac{E_{b}}{V_{b}}=\frac{(2\pi C_{l}+2C_{m})\gamma_{s}+\frac{HA}{3}\left(\frac{\cot\varphi_{e}}{\sqrt{3}}+1\right)+2\mu_{f}\sqrt{h_{i}(2R-h_{i})}\left(\frac{2s}{3\tan\theta_{f}}\right)\left(k_{1}H\sqrt{\frac{H}{E}}\right)}{0.5\pi C_{l}^{2}}$$

According to Equations (1)–(19), the specific cutting energy curves for ductile and brittle modes varying with the UCT *h_i_* can be simulated, which are related to the material properties, tool geometries, and cutting parameters. Arif et al. [26] have declared that the UCT *h_c_* could be predicted by the intersection point of two cutting energy curves. A pretty small range of the UCT is sufficient to predict the UCT *h_c_*, ranging from zero to a few tens or hundreds of nanometers, which is much less than the range of 0 to *fx_m_*/*R*, according to Equation (1). Moreover, the specific cutting energy curve for brittle mode can only be calculated provided that the mean crack depth (median or lateral crack depth) is known. Therefore, the UCT *h_c_* is related to the mean crack depth after the material and cutting tool are determined, which can be expressed as:

(20) $$C_{m}\mathrm{or}C_{l}=F(h_{c})$$

where *F* is a fitting function based on a series of simulated mean crack depth *C_m_* or *C_l_* and the UCT *h_c_*. Xiao et al. [27] considered that the function *F* could be exponential for brittle materials. Assuming that the generation mechanisms of all cracks in the shoulder region are the same, the relationship between the mean crack depth and critical UCT is the same as that between the crack depth and corresponding instantaneous UCT, then:

(21) $$C_{mi}\mathrm{or}C_{li}=\left\{ \begin{array}{l} F(h_{i})\;\;\;\;\;\;\;\;\;\;\;\;h_{i}\geq h_{c}\\ 0\;\;\;\;\;\;\;\;\;\;\;\;\;\;\;\;\;\;\;\;h_{i}<h_{c} \end{array}\right.$$

where the BDT of the workpiece material is considered.

The material removal is mainly the process in which lateral cracks in the shoulder region extend to the workpiece surface. The relationship between the shoulder damage depth (which is different from the damage depth in the cutting surface) and corresponding UCT *h_i_* can be established by Equation (21). Then, all of the shoulder damage depths at all *h_i_* from *h_c_* to *h_m_* can be calculated with the material properties, tool geometries, and cutting parameters. It should be pointed out that the calculation of the shoulder damage depth was involved in our previous study, where the assumption is made that the cutting tool is categorized as a sharp indenter [18]. This requires that many adjustment coefficients need to be fitted in advance by a large number of experiments. Nevertheless, the calculation model based on the specific cutting energies is more general in this paper, which considers the spring back and BDT of the workpiece material.

## 3. Experimental Details

One ultra-precision polished ZnSe crystal (Φ25 × 1 mm) was prepared. To determine its material properties, an in situ nanomechanical test system (Hysitron) was used to carry out nanoindentation and nanoscratch tests on the crystal surface. A Berkovich diamond indenter was used. The indentation and scratch loads ranged from 2000 to 8000 μN. Both the loading time and unloading time were 10 s in the nanoindentation test. The elastic modulus and hardness of ZnSe were obtained by the Oliver–Pharr method. In the nanoscratch test, the indenter was firstly indented into the crystal within 5 s and then scratched on it with a displacement of 10 μm and speed of 0.25 μm/s.

**Figure 2:**
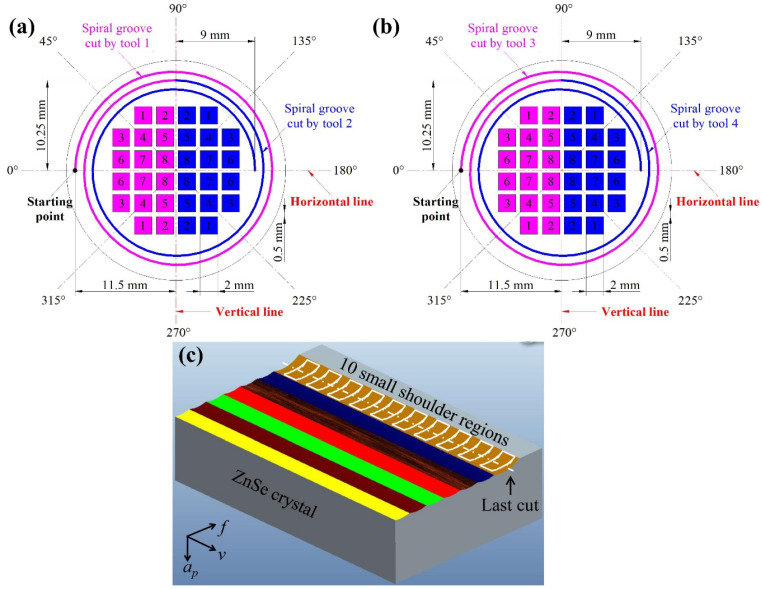
Schematic diagram of cutting experiments (**a**) by tools 1 and 2 on the front crystal surface, and (**b**) by tools 3 and 4 on the back crystal surface; (**c**) Processing procedure for each shoulder region (*f*: feed; *a_p_*: cutting depth; and *v*: cutting speed).

Next, a series of cutting experiments were conducted on the ZnSe crystal by the SPDT machine Moore Nanotech 350FG (Figure 1a). As shown in Figure 2a,b, two spiral grooves (i.e., pink and blue grooves) were scratched not only on the front crystal surface with tools 1 and 2 but also on the back crystal surface with tools 3 and 4. The values of the spindle speed, feed rate, and cutting depth are 3000 rev/min, 2 mm/min, and 1.0 μm, respectively. All cutting tools are new, and the geometric parameters are shown in Table 1. Both Figure 2a,b show the starting point of the spiral groove. Two groups of surfaces and shoulders, i.e., group A above and group B below the horizontal line, were obtained by orthogonal cutting. Within each group, there are sixteen cutting surfaces with corresponding shoulder regions. Among them, one half (on the left of vertical line) were cut by tool 1 (or tool 3) and the other half (on the right of vertical line) were cut by tool 2 (or tool 4). The cutting direction is along the vertical line. The size of each cutting surface is 2 mm × 2 mm, and the interval of the adjacent cutting surfaces is 0.5 mm. Figure 2c shows the processing procedure for each shoulder region. Each colored area corresponds to each cut, and the shoulder region produced by the last cut is researched. For orthogonal cutting experiments, the values or ranges of the cutting speed (*v*), feed (*f*), and cutting depth (*a_p_*) were correspondingly 800 mm/min, 0.5–5.0 μm/str, and 0.5–5.0 μm. The cutting parameters for groups A and B were established by random and optimal Latin hypercube designs, respectively, as shown in Table 2. The Zygo white light interferometer Newview 8300 was employed to measure the morphologies of the spiral grooves, shoulder regions, and cutting surfaces. Ten small shoulders were selected from each shoulder region (Figure 2c), the maximum damage depths for which were averaged as the final damage depth. Each cutting surface was cleaned with alcohol, acetone, and deionized water, respectively, for at least 5 min in an ultrasonic cleaning machine before each measurement.

## 4. Results and Discussions

### 4.1. Material Properties of ZnSe

**Figure 3:**
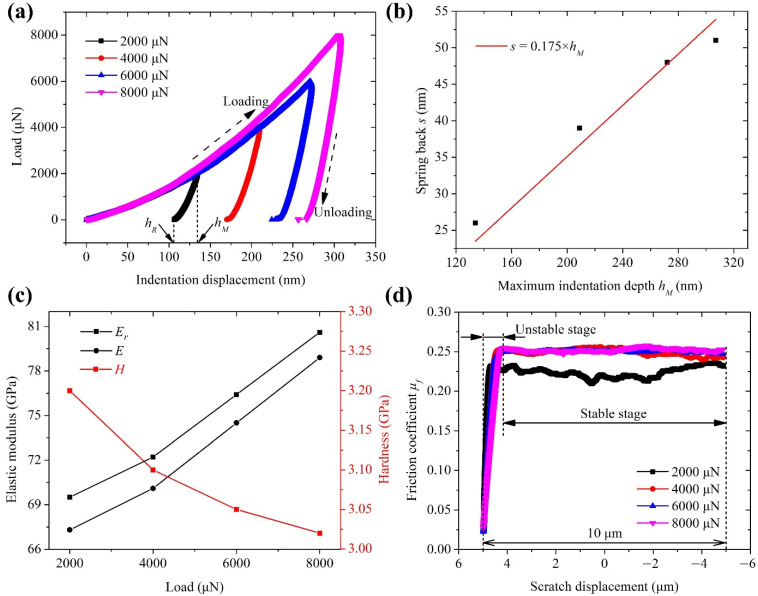
(**a**) Load-displacement curves under different indentation loads; (**b**) relationship between the spring back *s* and the maximum indentation depth *h_M_*; (**c**) reduced elastic modulus *E_r_*, elastic modulus, and hardness *H* of ZnSe under different indentation loads; (**d**) friction coefficient (*μ_f_*)-scratch displacement curves under different scratch loads.

Figure 3a shows load-displacement curves under different indentation loads. Each curve is divided into a loading stage and unloading stage. The displacement increases with the load during the loading stage. The abscissa at the end of the loading stage is the maximum indentation depth *h_M_*, and that at the end of the unloading stage is the residual depth *h_R_*. The value of *h_R_* is lower than that of the *h_M_* due to the elastic recovery of the workpiece material. According to the load-displacement curves, the *h_M_* = 134, 209, 272, and 307 μm, and the *h_R_* = 108, 170, 224, and 256 μm, under the loads of 2000, 4000, 6000, and 8000 μN, respectively.

According to Equation (11), it can be obtained as follows:

(22) $$s=k_{2}h_{M}$$

where the spring back *s* can be calculated by:

(23) $$s=h_{M}-h_{R}$$

Figure 3b shows the relationship between the spring back *s* and the maximum indentation depth *h_M_*, and their fitting result is *s* = 0.175 × *h_M_*. Based on the load-displacement curves, the reduced elastic modulus *E_r_* and hardness *H* of ZnSe under different indentation loads are measured and shown in Figure 3c. On this basis, the elastic modulus *E* of ZnSe is calculated by Equation (24) [28]:

(24) $$\frac{1}{E_{r}}=\frac{1-\nu^{2}}{E}+\frac{1-\nu_{in}^{2}}{E_{in}}$$

where *ν* is the Poisson’s ratio of ZnSe (*ν* ≈ 0.3), and ν*_in_* and *E_in_* are the Poisson’s ratio and elastic modulus of the diamond indenter (*ν_in_* = 0.07, *E_in_* = 1140 GPa), respectively. The calculated results are shown in Figure 3c. The average values of *E* and *H* are 72.7 GPa and 3.1 GPa, respectively, which are used for the model. Figure 3d shows the friction coefficient (*μ_f_*)-scratch displacement curves under different scratch loads. Each curve is divided into an unstable stage and stable stage. The unstable stage is generated since the indenter is firstly indented into the material and then scratched on it. The value of *μ_f_* in the stable stage is averaged as about 0.24 for the model, which is within the range of 0.2 to 0.4 reported by Venkatachalam et al. [19].

### 4.2. Critical UCT of ZnSe

**Figure 4:**
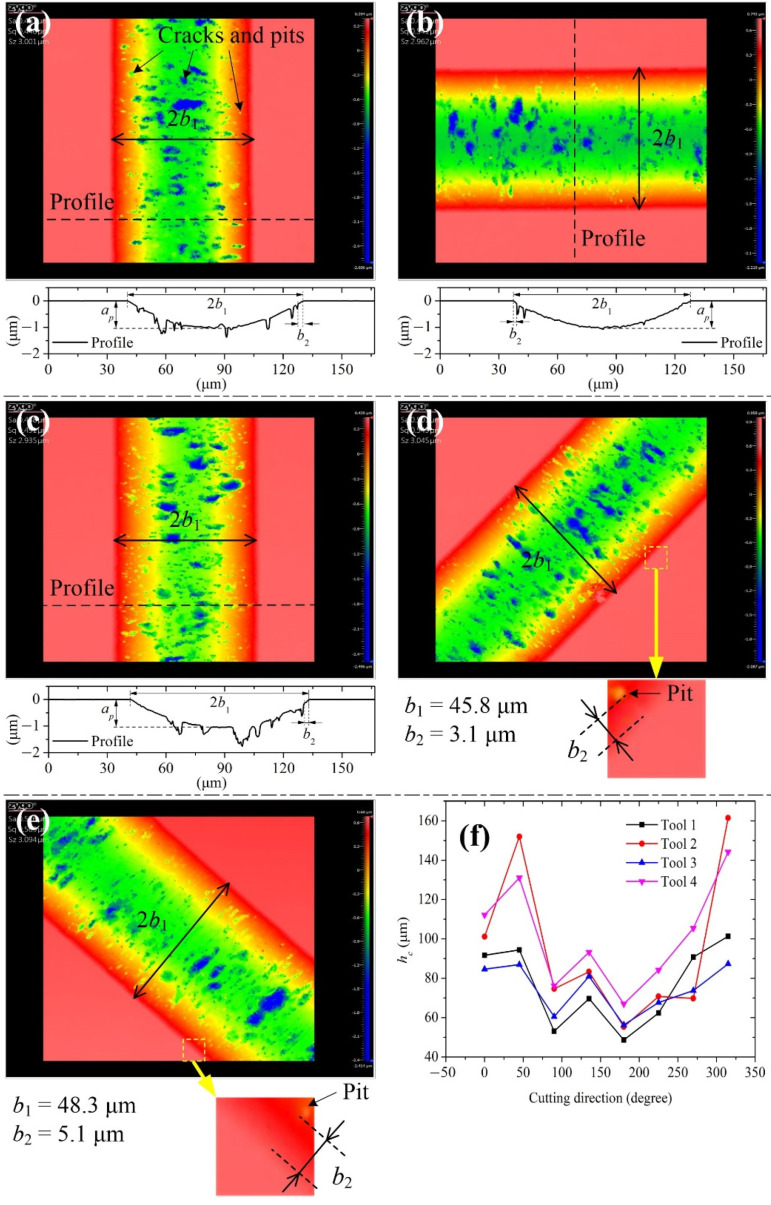
Morphology and profile of the groove under (**a**) 0°, (**b**) 90°, and (**c**) 180° cutting directions; morphology of the groove under (**d**) 45° and (**e**) 135° cutting directions; (**f**) different cutting directions induced by varying the critical UCT *h_c_*.

Spiral scratching is used to obtain the BDT morphology and critical UCT of ZnSe crystal under different cutting directions. Figure 2a,b show the cutting directions described by angle (0°, 45°, 90°, etc.). Figure 4a–c show the grooves with 0°, 90°, and 180° cut by tool 1, respectively. It can be observed that many large crater-like cracks occur in the middle of the groove, while small scattered micro-pits occur in the side of the groove. The damages significantly decrease at the edge of the groove. This is because the UCT in the middle of the groove is the largest, while it reduces toward the side of the groove. A scattered micropit is the major damage form below the small UCT. Smooth and rough areas coexist in the middle of the groove, indicating that the brittle and ductile cutting modes occur simultaneously. A profile across the groove is selected. Figure 4a–c show the same groove depths, about 1.0 μm (i.e., *a_p_*), indicating a flat machined surface. Chen et al. [29] have derived the equation of the critical UCT *h_c_* for spiral scratching:

(25) $$h_{c}=R\left[1-\frac{R-a_{p}}{\sqrt{R^{2}-{\left(b_{1}-b_{2}\right)}^{2}}}\right]$$

where *b*_1_ is the half-width of the groove; *b*_2_ is the minimum width of the ductile area. According to the groove profile or morphology, parameters *b*_1_ and *b*_2_ can be measured. For example, if *b*_1_ = 45.2 μm and *b*_2_ = 2.5 μm in Figure 4a, then *h_c_* = 91.7 μm; if *b*_1_ = 45.8 μm and *b*_2_ = 3.1 μm in Figure 4d, then *h_c_* = 94.3 μm. Figure 4f shows different cutting directions induced by the critical UCT *h_c_*. The UCT *h_c_* ranges from 48.6 nm to 101.3 nm for tool 1, from 55.3 nm to 161.5 nm for tool 2, from 56.3 nm to 87.3 nm for tool 3, and from 66.9 nm to 144.2 nm for tool 4. 

### 4.3. Shoulder Damage of ZnSe

**Figure 5:**
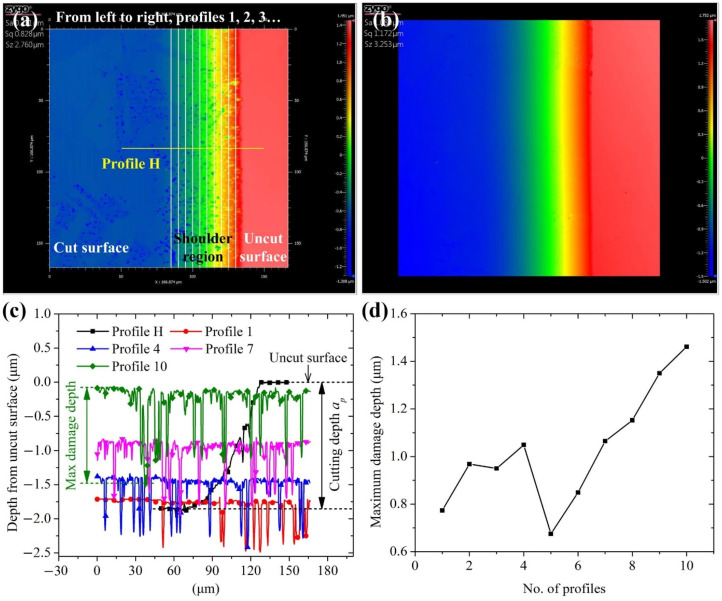
Damage morphologies in shoulders (**a**) 3 and (**b**) 5 cut by tool 1 under group A; (**c**) representative profiles from (**a**); (**d**) maximum damage depths for ten profiles shown.

Figure 5a,b show the damage morphologies in shoulders 3 and 5 cut by tool 1 under group A of the cutting parameters, respectively. Almost no damage occurs in shoulder 5, while many random fractures and micropits exist in shoulder 3. This can be explained by Equation (1) revealing that the larger *f* is, the higher *h_i_* may be at the same *x* coordinate, resulting naturally in a larger damage density and size. Shoulder 5 may be cut in ductile mode because the maximum UCT is less than critical UCT, i.e., *h_c_* > *fR*/*x_m_*. Figure 5a shows ten vertical profiles (profiles 1, 2, 3, etc.) distributed uniformly in shoulder 3 and one horizontal profile (profile H) across the uncut and cutting surfaces. The representative profiles are shown in Figure 5c. The cutting depth can be obtained from profile H, about 1.8 μm. The maximum damage depth for each vertical profile can be measured, as shown in Figure 5d. It can be found that the maximum damage depth generally increases with the number of profiles, or the instantaneous UCT. This is consistent with the findings from Figure 4. The groove bottom with the larger UCT experiences more serious cracks than that with the smaller UCT. In this paper, the maximum damage depth from ten vertical profiles is used for one small shoulder, and the damage depth for each shoulder can be obtained by averaging the maximum damage depths for ten small shoulders, as shown in Table 3. It should be pointed out that the shoulder damage depth is measured in the vertical direction, and the lateral crack depth is evaluated theoretically perpendicular to the circular arc of the tool nose (see Figure 1b). Although such a difference exists, the difference is very small. This is because the tool nose radius is usually far larger than the feed and cutting depth. Meanwhile, enough vertical profiles are selected to evaluate the shoulder damage depth.

**Figure 6:**
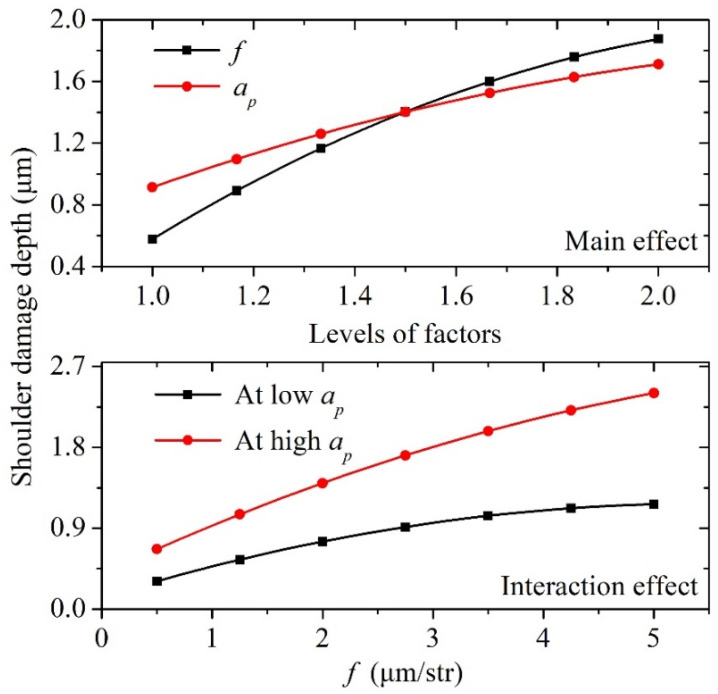
Effects of cutting parameters (feed *f*, cutting depth *a_p_*) on shoulder damage depth.

Using the measured shoulder damage depths shown in Table 3, the main and interaction effects of the cutting parameters on the shoulder damage depth can be discussed, as shown in Figure 6 [30]. The larger slope of the main effect curve indicates that the single factor feed *f* or cutting depth *a_p_* has a higher effect on the shoulder damage depth. A slope larger than zero means a positive effect; a slope smaller than zero denotes a negative effect; and a zero slope shows no effect. In the figure of the main effect, the horizontal axis represents the factor level: the values of 1.0 and 2.0 represent the low and high levels, respectively, corresponding to the minimum and maximum values of the cutting parameters in Table 3, respectively. It can be found that the damage depth increases as *f* or *a_p_* transits from low level to high level, and the effect of *f* on the damage depth is larger than that of *a_p_*. The non-parallel interaction effect curves indicate an interaction effect. Figure 6 shows that the effect of *f* on the damage depth at a high *a_p_* is larger than that at a low *a_p_*, and the interaction effect between *f* and *a_p_* is stronger at a smaller *f*.

### 4.4. Verification of Shoulder Damage Model

**Figure 7:**
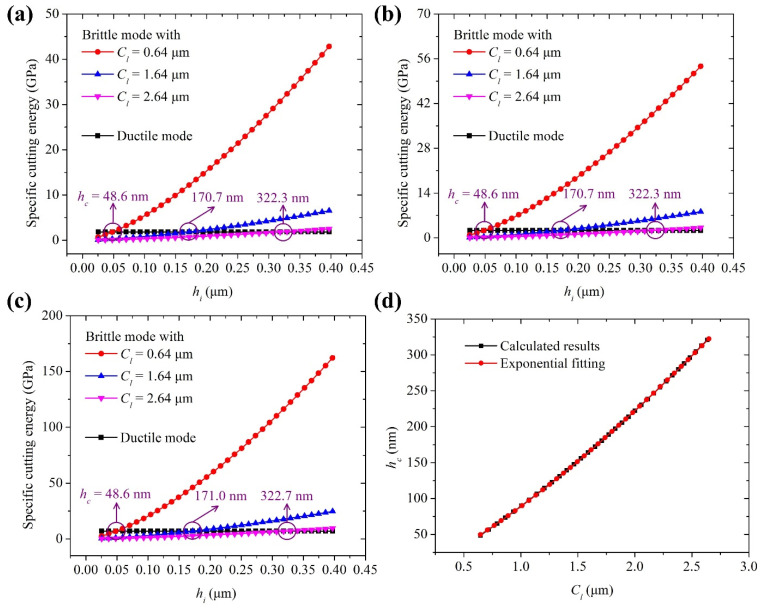
Variations of specific cutting energy with *h_i_* under different *C_l_* when (**a**) *k*_1_ = 0.41, (**b**) *k*_1_ = 4.1, and (**c**) *k*_1_ = 41; (**d**) relationship between *C_l_* and *h_c_* for tool 1 when *k*_1_ = 4.1. Note: *h_i_*: instantaneous UCT; *h_c_*: critical UCT; and *C_l_*: mean lateral crack depth.

Based on Equations (1)–(19), if the crack depth in the shoulder region is known, the specific cutting energies for ductile and brittle modes can be determined from the material properties and tool geometries, and then the critical UCT *h_c_* can be obtained. The parameter *k*_1_ in Equation (8) should be known in advance, although it has been set to 4.1 for these metal materials: Al6061-T6, Al5086, Cu, and Ni [22]. Figure 7a–c show the variations of the specific cutting energy with UCT *h_i_* under different lateral crack depths when *k*_1_ = 0.41, 4.1, and 41. It can be found that the two cutting energy curves for ductile and brittle modes intersect with each other, and the UCT *h_c_* occurs at the intersection point. It can be also found that the influence of the parameter *k*_1_ on the UCT *h_c_* is very small, which may be attributed to the different material properties with those metals. This does not mean that the parameter *k*_1_ shows no effect on the stress at the flank-workpiece interface and cutting force. The specific cutting energies in ductile and brittle mode increase with the value of *k*_1_ at the same time, while their curve intersection point position changes slightly. In the following analysis, *k*_1_ is set as 4.1. Figure 7d shows that the UCT *h_c_* increases nonlinearly with the lateral crack depth *C_l_* for tool 1, which well accords with the exponential equation, i.e., *h_c_* = 88.9$C_{l}^{1.322}$. Similarly, *h_c_* = 145.6$C_{l}^{1.410}$, *h_c_* = 189.9$C_{l}^{1.349}$, and *h_c_* = 223.1$C_{l}^{1.445}$ can be obtained for tools 2, 3, and 4, respectively. The relationship between the crack depth and corresponding instantaneous UCT is the same as that between the mean crack depth and critical UCT, which is expressed as:

(26) $$\begin{array}{l} C_{li}=\left\{ \begin{array}{l} 0.0336\times h_{i}^{0.756}\;h_{i}\geq 48.6\mathrm{nm}\\ 0\;\;\;\;\;\;h_{i}<48.6\mathrm{nm} \end{array}\right.\mathrm{for}\;\mathrm{tool}\;1\\ C_{li}=\left\{ \begin{array}{l} 0.0292\times h_{i}^{0.709}\;h_{i}\geq 55.3\mathrm{nm}\\ 0\;\;\;\;\;\;h_{i}<55.3\mathrm{nm} \end{array}\right.\mathrm{for}\;\mathrm{tool}\;2\\ C_{li}=\left\{ \begin{array}{l} 0.0205\times h_{i}^{0.741}\;h_{i}\geq 56.3\mathrm{nm}\\ 0\;\;\;\;\;\;h_{i}<56.3\mathrm{nm} \end{array}\right.\mathrm{for}\;\mathrm{tool}\;3\\ C_{li}=\left\{ \begin{array}{l} 0.0237\times h_{i}^{0.692}\;h_{i}\geq 66.9\mathrm{nm}\\ 0\;\;\;\;\;\;h_{i}<66.9\mathrm{nm} \end{array}\right.\mathrm{for}\;\mathrm{tool}\;4 \end{array}$$

**Figure 8:**
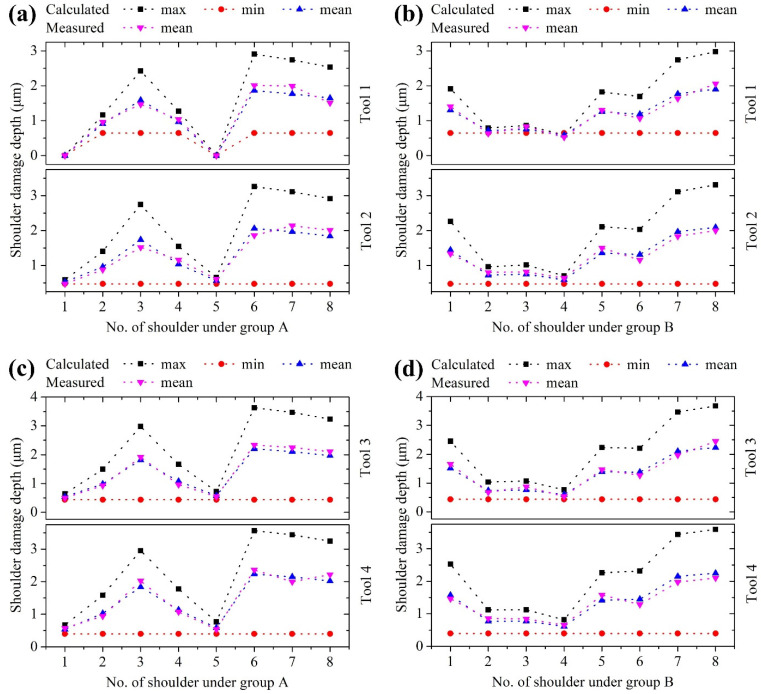
Measured and calculated shoulder damage depths under (**a**) group A and (**b**) group B using tools 1 and 2, and under (**c**) group A and (**d**) group B using tools 3 and 4.

The damage depth is related to a lateral crack in the shoulder region, which can be also estimated by Equation (26). Figure 8a–d show the measured and calculated shoulder damage depths. It can be found from Figure 8a that the calculated damage depths in shoulders 1 and 5 cut by tool 1 are zero. At this point, the cutting is ductile. The calculated minimum damage depths in the other shoulders remain 0.645 μm, corresponding to the lateral crack depth at the BDT zone. It can be also observed that the calculated minimum damage depths in all shoulders cut by tools 2, 3, and 4 are the same as 0.473, 0.437, and 0.396 μm, respectively. The relative error between the measured and calculated values is defined as:

(27) $$\mathrm{relative}\;\mathrm{error}=\frac{\left|\mathrm{measured}\;\mathrm{value}-\mathrm{calculated}\;\mathrm{value}\right|}{\mathrm{measured}\;\mathrm{value}}\times 100\%$$

According to Equation (27), the maximum relative errors are calculated as 12.55%, 13.81%, 12.66%, and 12.58% for tools 1, 2, 3, and 4, respectively. The average relative errors are 6.01%, 6.47%, 6.26%, and 6.29% for tools 1, 2, 3, and 4, respectively. The error sources are analyzed as follows: (1) There is a small difference between the shoulder damage depth measured in the vertical direction and the lateral crack depth evaluated perpendicular to the circular arc of the tool nose; (2) the shoulder damage depth is measured by a Zygo optical profiler, which usually has transmission and reflection problems during the measurement of depth; (3) the spring back and friction coefficient are measured by nanoindentation and nanoscratch tests, respectively, where the indenter is different from the cutting tool, and the indentation/scratch process has a certain difference from the cutting process; and (4) the values of some parameters are obtained empirically, such as the cutting ratio *r_c_* and specific surface energy *γ_S_*. Although there are so many error sources, the average relative error is smaller than 7%, indicating the model’s feasibility. Since Latin hypercube designs have a strong random characteristic and a great filling capacity, the shoulder damage model has nice applicability and generality. The proposed modeling method will be beneficial to the ultra-precision diamond machining of other brittle materials.

### 4.5. Application of Shoulder Damage Model

**Figure 9:**
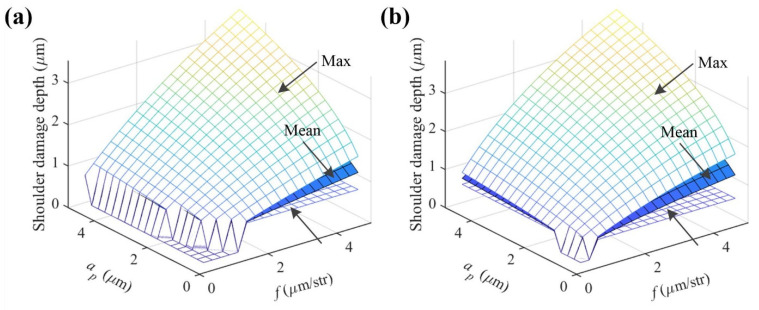

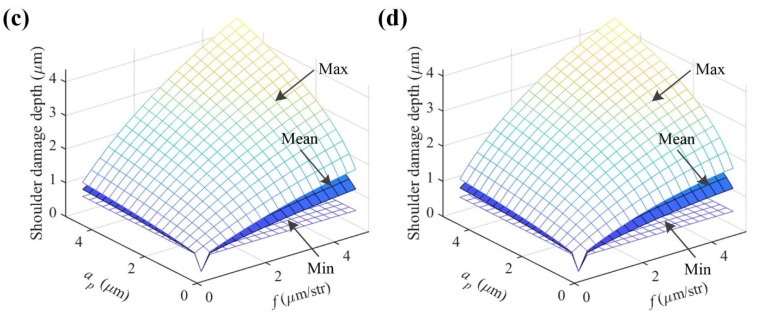
Calculated shoulder damage depths under a broader range of cutting parameters using (**a**) tool 1, (**b**) tool 2, (**c**) tool 3, and (**d**) tool 4.

**Figure 10:**
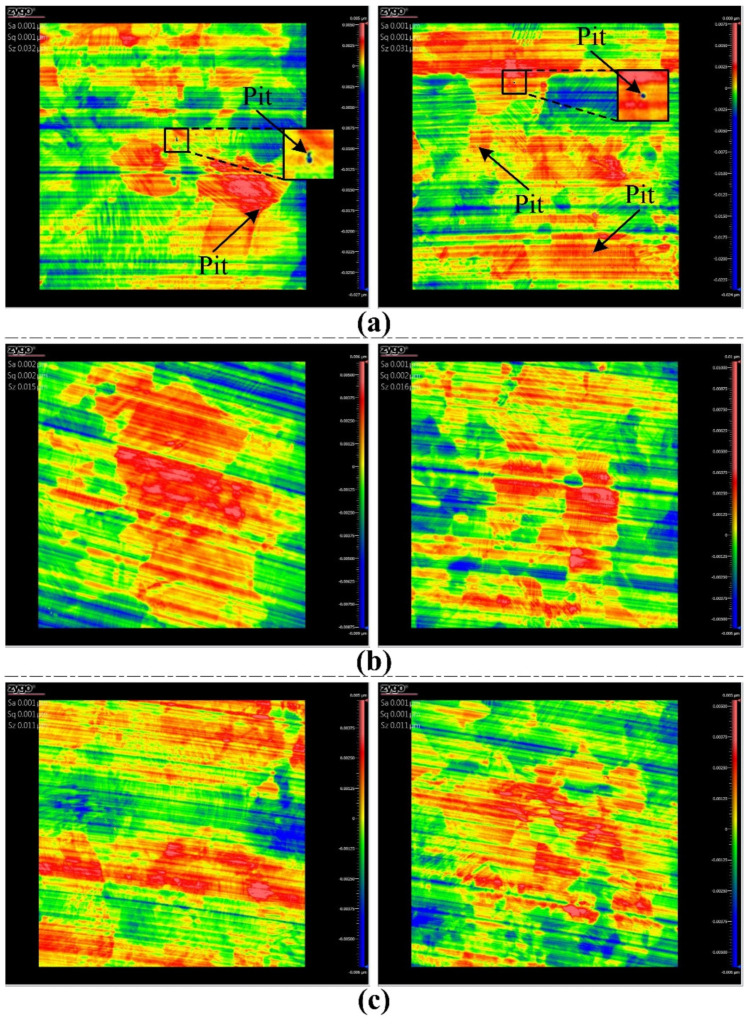
Surface morphologies cut by tool 1 at (**a**) 1.8 mm/min (*f* = 0.6 μm/rev), (**b**) 1.5 mm/min (*f* = 0.5 μm/rev), and (**c**) 1.2 mm/min (*f* = 0.4 μm/rev) feed rates under the same spindle speed (3000 rev/min) and cutting depth (2.5 μm).

The model of the shoulder damage can be used to providethe critical cutting parameters necessary to achieve a smooth surface. When the UCT *h_i_* is smaller than the critical value *h_c_*, no damage occurs in the shoulder region, resulting in a cutting surface without any cracks or pits. The shoulder damage depth is calculated under a broader range of cutting parameters, as shown in Figure 9a–d. There is no damage occurring under the feed *f* ≤ 0.5 μm/str and *a_p_* ≤ 4.75 μm or feed *f* ≤ 0.75 μm/str and *a_p_* ≤ 2.0 μm using tool 1, under the feed *f* ≤ 0.5 μm/str and *a_p_* ≤ 1.25 μm or feed *f* ≤ 0.75 μm/str and *a_p_* ≤ 0.5 μm using tool 2, or under the feed *f* ≤ 0.5 μm/str and *a_p_* ≤ 0.5 μm using tools 3 or 4. This indicates that tool 1 has the best processing performance, which may be attributed to the fact that tool 1 has the largest tool nose radius. Thus, several groups of the critical cutting parameters are obtained to achieve a smooth surface without any micropits, the major damage form. Here, a group of the critical cutting parameters is validated by spiral cutting. The reason for choosing spiral cutting is that it has the advantage of reducing figure error. Three ZnSe crystals (Φ25 × 1 mm) are cut by tool 1 at 1.8 mm/min (*f* = 0.6 μm/rev), 1.5 mm/min (*f* = 0.5 μm/rev), and 1.2 mm/min (*f* = 0.4 μm/rev) feed rates respectively under the same spindle speed (3000 rev/min) and the cutting depth (2.5 μm), which are often used in the actual diamond turning. It should be noted that the unit of feed rate is μm/str in orthogonal cutting, while it changes to μm/rev in spiral cutting. Figure 10 shows the final cutting surfaces with an area of 0.167 × 0.167 mm^2^. It can be found from Figure 10a (*f* = 0.6 μm/rev) that small micro-pits distribute randomly, whereas from Figure 10b,c (*f* = 0.5 μm/rev, 0.4 μm/rev) it can be found that no micropit occurs. The values of roughness Sz are ~32 nm, ~16 nm, and ~11 nm, respectively, and the corresponding roughness valley values are ~26 nm, ~8 nm, and ~6 nm. All roughness Sa values are 1 nm. It should be pointed out that the smooth surface here may still have some nanodefects, while it can completely meet the requirement for the infrared optical image systems [31].

**Figure 11:**
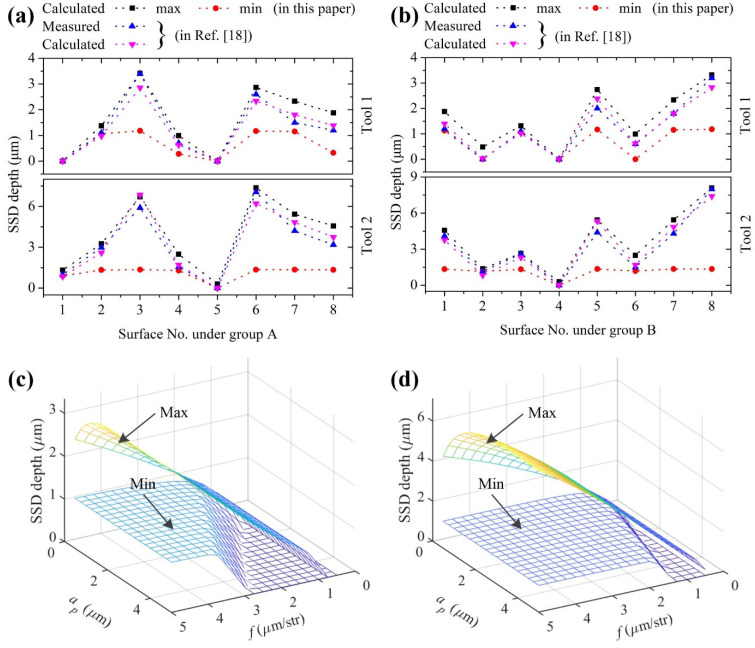
Measured and calculated SSD depths under (**a**) group A and (**b**) group B using tools 1 and 2; calculated SSD depths using (**c**) tool 1 and (**d**) tool 2 under a wider range of cutting parameters.

This model of the shoulder damage can be also used to predict the bound of the SSD depth. For diamond cutting ZnSe crystal, Xiao et al. [18] have calculated the SSD depth with an average relative error of less than 15.0% and a maximum relative error up to 20.0%, as shown in Figure 11a,b. Meanwhile, nearly half of the calculated values are smaller than the measured ones, making it difficult to determine the material removal volume during subsequent processes. Therefore, it is necessary to predict the bound of the SSD depth. The SSD would appear if the median crack in the shoulder region penetrates the cutting surface. As shown in Figure 1b, in triangle O_1_O_2_B*_i_*, there is:

(28) $$\cos\omega_{i}=\frac{{\left(R-h_{i}\right)}^{2}+f^{2}-R^{2}}{2f(R-h_{i})}$$

where *ω_i_* is the transition angle at each *h_i_*. Considering the BDT of the workpiece and assuming the SSD*_i_* represents the SSD depth at *h_i_*, SSD*_i_* can be established as:

(29) $${\mathrm{SSD}}_{i}=\left\{ \begin{array}{l} (R-h_{i}+C_{mi})\sin\omega_{i}-R\;h_{i}\geq h_{c}\\ 0\;\;\;\;\;\;\;\;\;\;h_{i}<h_{c} \end{array}\right.$$

All SSD*_i_* at all *h_i_* can be predicted by Equation (29), and then the bound of the SSD depth can be determined. Figure 11a shows the maximum and minimum SSD depths under group A using tools 1 and 2 with the material properties in [18]. It can be found that all measured SSD depths are between the calculated maximum and minimum values, and closer to the maximum values, which is also verified by Figure 11b. The bound of the SSD depth is predicted under a wider range of cutting parameters, as shown in Figure 11c,d. The maximum SSD depth generally increases with feed or cutting depth. No SSD occurs under the feed *f* ≤ 0.75 μm/str using tool 1, and the SSD always occurs under the feed 0.5 ≤ *f* ≤ 5.0 μm/str using tool 2.

## 5. Conclusions

Based on the specific cutting energies for brittle and ductile mode machining, the model of the shoulder damage is proposed for ultra-precision diamond cutting of ZnSe crystal. Based on the measured material properties, critical UCT, and shoulder damage depth of ZnSe crystal, the model is validated by experiments. The model is used to provide the critical cutting parameters to achieve a smooth surface and to predict the SSD depth bound of a cutting surface by considering the kinetic characteristics of SPDT.

The detailed conclusions are listed as follows:

(1) The shoulder damage depth has a positive correlation with the instantaneous undeformed chip thickness, which increases with the feed or cutting depth. The effect of the feed on the shoulder damage depth at a high cutting depth is larger than that at a low one. Moreover, the interaction effect is especially obvious when the feed is small.
(2) The shoulder damage depth and the SSD depth bound of a cutting surface can be evaluated effectively. The shoulder damage model has an average relative error of less than 7%. The upper bound of the SSD depth generally increases with the feed or cutting depth.
(3) A smooth ZnSe surface with roughness Sa = 1.0 nm is machined by SPDT.

Future research will focus on combining more influence factors into the model of the shoulder damage, especially tool wear and waviness, workpiece vibration, and deformation. To achieve nanodefect-free machining of ZnSe optical elements, a comprehensive characterization of nanodamages will also be a research focus.

## Figures and Tables

**Table 1:** Geometry parameters of cutting tool.

Tool No.	Nose Radius *R* (mm)	Nominal Rake Angle *α* (°)	Clearance Angle *θ_f_* (°)	Cutting Edge Radius *r* (nm)
1	1.004	0	12	≈25
2	0.201	−25	12	≈20
3	0.098	0	15	≈20
4	0.052	−25	12	≈15

**Table 2:** Groups A and B of cutting parameters (*f*: feed; *a_p_*: cutting depth).

No. of Cutting Surface or Shoulder Region	Group A		Group B	
	*f* (μm/str)	*a_p_* (μm)	*f* (μm/str)	*a_p_* (μm)
1	1.1	0.5	3.1	2.4
2	2.4	1.1	1.1	1.8
3	5.0	1.8	2.4	0.5
4	1.8	2.4	0.5	3.7
5	0.5	3.1	4.4	1.1
6	4.4	3.7	1.8	5.0
7	3.7	4.4	3.7	4.4
8	3.1	5.0	5.0	3.1

**Table 3:** Measured shoulder damage depths for each shoulder.

No. of Shoulder	Damage Depth under Group A (μm)				Damage Depth under Group B (μm)			
	Tool 1	Tool 2	Tool 3	Tool 4	Tool 1	Tool 2	Tool 3	Tool 4
1	≈0.00	0.47	0.49	0.57	1.41	1.34	1.66	1.46
2	0.96	0.88	0.93	0.94	0.63	0.80	0.68	0.86
3	1.47	1.52	1.92	2.03	0.83	0.82	0.87	0.84
4	1.04	1.16	0.96	1.07	0.52	0.64	0.54	0.66
5	≈0.00	0.61	0.54	0.54	1.30	1.50	1.47	1.58
6	2.01	1.86	2.34	2.36	1.07	1.16	1.27	1.29
7	1.99	2.14	2.25	2.00	1.63	1.83	1.97	1.97
8	1.51	2.01	2.11	2.22	2.06	2.00	2.45	2.10

## Data Availability

Not applicable.
